# Dexamethasone PLGA Microspheres for Sub-Tenon Administration: Influence of Sterilization and Tolerance Studies

**DOI:** 10.3390/pharmaceutics13020228

**Published:** 2021-02-06

**Authors:** Deyanira Barbosa-Alfaro, Vanessa Andrés-Guerrero, Ivan Fernandez-Bueno, María Teresa García-Gutiérrez, Esther Gil-Alegre, Irene Teresa Molina-Martínez, José Carlos Pastor-Jimeno, Rocío Herrero-Vanrell, Irene Bravo-Osuna

**Affiliations:** 1 Innovation, Therapy and Pharmaceutical Development in Ophthalmology (InnOftal) Research Group, UCM 920415, Complutense University of Madrid, 28040 Madrid, Spain; deyanira.barbosa@hotmail.com (D.B.-A.); vandres@ucm.es (V.A.-G.); megil@ucm.es (E.G.-A.); iremm@ucm.es (I.T.M.-M.); ibravo@ucm.es (I.B.-O.); 2 Departamento de Farmacia Galénica y Tecnología Alimentaria, Facultad de Farmacia, Universidad Complutense de Madrid (UCM), IdISSC, 28040 Madrid, Spain; 3 Thematic Research Network in Ophthalmology (Oftared) Carlos III National Institute of Health, 28040 Madrid, Spain; ivan.fernandez.bueno@uva.es (I.F.-B.); pastor@ioba.med.uva.es (J.C.P.-J.); 4 Instituto Universitario de Oftalmobiología Aplicada (IOBA), Universidad de Valladolid, 47011 Valladolid, Spain; tgarcia@ioba.med.uva.es; 5 Department of Ophthalmology, Hospital Clínico Universitario of Valladolid, 47003 Valladolid, Spain

**Keywords:** dexamethasone, poly(lactic-*co*-glycolic) acid, ophthalmology, microspheres, periocular administration, gamma sterilization, tolerance

## Abstract

Many diseases affecting the posterior segment of the eye require repeated intravitreal injections with corticosteroids in chronic treatments. The periocular administration is a less invasive route attracting considerable attention for long-term therapies. In the present work, dexamethasone-loaded poly(lactic-*co*-glycolic) acid (PLGA) microspheres (Dx-MS) were prepared using the oil-in-water (O/W) emulsion solvent evaporation technique. MS were characterized in terms of mean particle size and particle size distribution, external morphology, polymer integrity, drug content, and in vitro release profiles. MS were sterilized by gamma irradiation (25 kGy), and dexamethasone release profiles from sterilized and non-sterilized microspheres were compared by means of the similarity factor (f_2_). The mechanism of drug release before and after irradiation exposure of Dx-MS was identified using appropriate mathematical models. Dexamethasone release was sustained in vitro for 9 weeks. The evaluation of the in vivo tolerance was carried out in rabbit eyes, which received a sub-Tenon injection of 5 mg of sterilized Dx-MS (20–53 µm size containing 165.6 ± 3.6 µg Dx/mg MS) equivalent to 828 µg of Dx. No detectable increase in intraocular pressure was reported, and clinical and histological analysis of the ocular tissues showed no adverse events up to 6 weeks after the administration. According to the data presented in this work, the sub-Tenon administration of Dx-MS could be a promising alternative to successive intravitreal injections for the treatment of chronic diseases of the back of the eye.

## 1. Introduction

According to the World Health Organization, there are 2.2 billion people globally who have a vision impairment or blindness. Of this, available data suggest a conservative estimate of at least one billion people with moderate or severe distance vision impairment or blindness that could have been prevented or has yet to be addressed. This number includes those with moderate or severe distance vision impairment or blindness due to unaddressed refractive error (123.7 million), cataract (65.2 million), age-related macular degeneration (10.4 million), glaucoma (6.9 million), corneal opacities (4.2 million), diabetic retinopathy (3 million), and trachoma (2 million), as well as near-vision impairment caused by unaddressed presbyopia (826 million). The estimate is based on recently published epidemiological data on the global magnitude of near-vision impairment [1] and the global magnitude and causes of bilateral distance vision impairment and blindness [2]. With such a large number of cases each year, it is critical that an optimal treatment is used in ophthalmic chronic diseases. 

Many diseases affecting the posterior segment of the eye, such as macular edema secondary to diabetic retinopathy, to central and branch retinal vein occlusion, after surgery (retinal detachment or epiretinal membrane peeling, Irvine–Gass syndrome (post cataract surgery), or after posterior uveitis, require long-term treatments with corticosteroids. Since it is particularly difficult to achieve effective levels of therapeutics in the vitreous/retina following a topical or systemic administration, intraocular drug delivery devices that are capable of maintaining effective intravitreal levels for long periods, with minimal systemic exposure, have attracted considerable attention [3,4,5,6]. Of special interest is the design of intravitreal biodegradable implants that can be placed into the vitreous without the need for surgical procedures and without the need to be removed later on. Their development represents a significant advance because they are able to maintain therapeutic concentrations of drugs in the vitreous for long periods (from days to months) [7,8]. However, they present problems such as the ability to migrate to the anterior segment in pseudophakic patients, or the management of secondary ocular hypertensions. An intravitreal biodegradable implant loaded with dexamethasone (Dx) (0.7 mg) is currently in the clinical practice marketed for the treatment of macular edema due to retinal vein occlusion and noninfectious uveitis (Ozurdex™; Allergan Inc., Irvine, CA, USA) [9,10,11,12]. 

In addition to drug implants, other formulations capable of achieving sustained intraocular therapeutic drug concentrations are being studied, for example, microparticles made up of biodegradable polymers, injected as an aqueous suspension in a periocular or intraocular fashion to achieve sustained drug levels [13,14]. Biodegradable poly(lactic-*co*-glycolic) acid (PLGA) microspheres form a depot after their injection acting as an in situ biodegradable implant [15]. The main advantage of microspheres is that doses and specific combination therapy can be easily tuned for each patient as different doses of active substances could be administered by changing the amount of microspheres.

Considering the administration route, the most effective way to achieve appropriate drug concentrations in the posterior eye segment is via intravitreal injection. However, intravitreal administration has potential severe side-effects such as retinal detachment and endophthalmitis [16]. Furthermore, the risk of secondary adverse events increases with the number of injections. Some treatments, for example antiangiogenic treatments at the retinal level, are based on intravitreal injection of anti-vascular endothelial growth factor (anti-VEGF) active compounds once a month for life; thus, with time, these injection side-effects become another problem to deal with from a clinical point of view, in addition to the disease itself.

A potentially less invasive alternative is periocular administration, such as the retrobulbar, subconjunctival, and posterior sub-Tenon injections. Periocular administrations routes are being explored to avoid the mentioned problem, with the studies performed in the case of corticosteroid administration being especially relevant [17]. Among the periocular routes explored, the sub-Tenon seems suitable to treat the deep zones of the back of the eye and it could be an alternative to intravitreal injections, especially with respect to the administration of polymeric drug delivery systems, able to release the loaded active compound for months in a sustained manner. It is important to remark the advantages of the sub-Tenon administration, which is adjacent to the sclera and delivers the drug directly into the retina and vitreous. The sclera is a highly hypovascular porous fibrous tissue (16 nm effective pore size) [18] with low blood absorption and high permeability, and it offers a large surface and a large volume of injection (up to 5 mL) [19], if compared with the vitreous cavity, whose volume of injection is limited to 20–200 µL. After scleral permeation, the compounds reach the ciliary body anteriorly and the choroid posteriorly. For retinal delivery, drugs also need to be able to permeate across the retinal pigment epithelium, which forms an effective cellular barrier due to tight junctions between the retinal pigment epithelium (RPE) cells. In addition to these barriers, the feasibility of drugs to access the vitreous after periocular administration has been reported. For example, according to experimental studies in rabbit eyes, the administration of dimethyl vancomycin via sub-Tenon administration provided an effective drug concentration in the vitreous [20]. Furthermore, previous studies have shown that, after peribulbar or subconjunctival injection of dexamethasone, significant levels of the active substance can be measured in the vitreous, and these levels are achieved by direct diffusion of the drug through the sclera [21]. For example, in a very interesting approach, Huang et al. [22] evaluated some pharmacokinetic parameters of dexamethasone sodium phosphate (DSP) in the rabbits’ posterior segment tissue following, among others, sub-Tenon perfusion showing t_½_ values for DSP of 14.32 h, 7.65 h, and 4.89 h for vitreous, choroidal/retinal compound, and sclera, respectively. Three years later, the same group demonstrated the efficacy of the sub-Tenon infusion of DSP in experimental autoimmune uveitis in rabbits [23]. For large molecules, these routes have shown a slower diffusion rate or less sustained effect relative to intravitreal injections. In humans, an anti-inflammatory and anti-infective response was reported in a limited phase II study, in which biodegradable microspheres containing triamcinolone acetonide and ciprofloxacin were administered via sub-Tenon injection, in a small group of patients after cataract extraction and intraocular lens implantation [24]. Considering this, sub-Tenon administration provides new possibilities to evaluate DDS as a new tool that is able to prolong a drug’s efficacy in the posterior segment of the eye, considerably reducing the number of interventions by the ophthalmologist and, subsequently, limiting the risks associated, especially in chronic treatments. Moreover, this route can be exploited for long-term treatment without serious side-effects and potential interference with the optical path [25].

From a technological point of view, an essential issue in the development of formulations intended for ophthalmic administration is the sterilization process. The use of gamma-irradiation is the most suitable sterilization method for PLGA-based microparticles [15,26,27]. However, this radiation method can have effects on the biodegradable polyesters, i.e., the formation of reactive radicals, which may compromise the drug substance incorporated into the device. Therefore, drug stability under irradiation conditions and changes in drug release properties need to be carefully evaluated [28,29].

The purpose of this investigation was to develop a sterilized formulation of dexamethasone-loaded PLGA microspheres (Dx-MS) able to control the release of the drug, while being at the same time well tolerated after sub-Tenon administration. To that, microspheres were designed, prepared, and characterized in vitro. Then, microspheres were injected periocularly (sub-Tenon administration) into rabbit eyes to evaluate the tolerance of the formulation in terms of clinical evaluation and histological analysis.

## 2. Materials and Methods 

Poly(d,l-lactide-*co*-glycolide) (PLGA, ratio 50:50; Resomer^®^ RG 502) was purchased from Boehringer Ingelheim (C.H. Boehringer Sohn AG & Ko. KG, Ingelheim am Rhein, Germany). Polyvinyl alcohol (PVA, 67,000 g/mol) was supplied by Merck KGaA (Darmstadt, Germany). Dexamethasone (Dx, M_W_ 392.5 g/mol; aqueous solubility at 37 °C = 0.12 mg/mL) was obtained from Sigma-Aldrich (Schnelldorf, Darmstadt, Germany). All other chemicals were reagent grade and used as received.

### 2.1. Preparation of Microspheres

Dx-loaded PLGA microspheres (Dx-MS) were prepared using the oil-in-water (O/W) emulsion solvent evaporation technique as previously described [30]. Briefly, 80 mg of Dx was suspended in 0.8 mL of PLGA/dichloromethane (50% *w*/*v*) by ultrasonication in a water/ice bath for 5 min (Elma. Transsonic 460, Singen, Germany). Then, the suspension was gently sonicated at low temperature (Sonicator KL, Qsonica Llc, Newtown, CT, USA) for 1 min. The so-prepared organic phase was emulsified with 5 mL of PVA MilliQ^®^ water solution (1% *w*/*v*). Emulsification was performed in a homogenizer (Polytron^®^ RECO, Kinematica GmbH PT 3000, Lucerna, Switzerland) at different speeds (5500–9500 rpm) for 2 min. Subsequently, 10 mL of a PVA solution (0.1%) was added to the emulsion and kept in the same homogenization conditions for an extra minute. Finally, the emulsion was poured into 90 mL of an aqueous PVA solution (0.1%) and kept under constant stirring for 3 h at room temperature, to allow the extraction and the subsequent evaporation of the organic solvent. Once formed, microspheres were washed with MilliQ^®^ water to eliminate PVA and sieved according to their particle size (106–53 µm, 53–38 µm, 38–20 µm, or 20–2 µm). Particles were then rapidly frozen (methanol/ice mixture) and freeze-dried to obtain a free-flowing microsphere powder. They were kept at −20 °C under dry conditions until use. Three independent batches (same composition) of microspheres were prepared in each experimental condition (four different homogenization speeds). For each experimental condition, microspheres with sizes 53–38 µm and 38–20 µm were selected for further analysis. For the in vitro release profile, 10 different batches of the selected formulation were prepared, and the whole 53–20 fraction was selected. 

### 2.2. Microspheres Characterization

#### 2.2.1. Production Yield

The production yield percentage (*PY*%) of each size fraction was calculated from the following Equation (1):

(1) $$PY(\%)=\frac{M_{T}(microspheres)}{M_{T}(PLGA)+M_{T}(Dx)}\times 100,$$

where *M_T_* is the mass of each component in the formulation.

#### 2.2.2. Mean Particle Size, Particle Size Distribution, and Morphology of Microspheres

Mean particle size and particle size distribution were measured by static light scattering (Microtrac S3500, Montgomeryville, PA, USA). Samples were prepared by suspending microspheres in distilled water. Microsphere morphology was evaluated by scanning electron microscopy (SEM; Jeol, JSM-6335F, Tokyo, Japan). Prior to that, each powder sample was spread on a double-sided carbon tape mounted on an aluminum stub to be gold-coated under vacuum (K550X ion sputter, Emitech, UK) for 3 min at 25 mA. SEM images were recorded at an acceleration voltage of 5.0 kV and 2500× magnification.

#### 2.2.3. Polymer Integrity

The molar mass of the polymers was evaluated by high-performance gel permeation chromatography (GPC). The assay was performed with a blank column of PLgel (PLgel 3 µm MIXED-E and PLgel 5 µm MIXED-D, both 7.8 mm × 300 mm), preceded by a PLgel 5 µm guard, 50 mm × 7.5 mm from Varian (Polymer Laboratories, Church Stretton, Shropshire, UK). The equipment included a Waters 1525 binary HPLC pump and a Waters 2414 Refractive Index Detector (Waters, Saint-Quentin en Yvelines, France). The column was maintained at 33 °C using a Waters heating column system. Tetrahydrofuran (THF) was filtered (Sartolon Polyamid, 0.45 µm, Göttingen, Germany) and degassed before use. The flow rate was 1 mL/min of THF. Before injection, samples were filtered using a polytetrafluoroethylene (PTFE) membrane (0.22 µm, 0.2 mm × 13 mm, Teknokroma, Barcelona, Spain). The injection volume was 20 µL (Hamilton syringe, Reno, NV, USA). 

The column was calibrated using polystyrene standards of different molar masses: 381, 1100, 2950, 6520, 18,600, and 43,700 g/mol (Waters, Mainz, Germany), prepared by their dissolution in THF at a concentration ranging from 0.4 to 1.6 mg/mL, depending on the molar mass needed. Experimental samples were dissolved in THF at concentrations ranging from 1 to 2 mg/mL.

#### 2.2.4. Drug Quantification by HPLC

Dexamethasone was quantified by HPLC, according to the method described in the Spanish Royal Pharmacopoeia, with minor modifications. The method was validated with respect to linearity, accuracy, and reliability in the range of concentrations of 2–20 µg/mL. 

The HPLC system consisted of two Waters Millipore 510 pumps, an autosampler Waters 712DWSP, and a Waters 490E ultraviolet/visible light (UV/Vis) detector. Analyses were performed on a 15 cm × 5 mm reverse-phase C18 5-µm column (Tracer Excel 120 ODSA, Teknokroma, Barcelona, Spain) preceded by a guard cartridge SEA 18, 100 mm × 4.0 mm (Teknokroma, Barcelona, Spain). The column was maintained at 45 °C using a heating column system (Waters TCM/CMM). The mobile phase consisted on a gradient of acetonitrile–water 35:65 *v*/*v* (phase A) and acetonitrile (phase B) delivered at a flow rate of 1.0 mL/min. The initial mobile phase condition A 100%‒B 0% was progressively changed to A 0%‒B 100% from *t* = 10 min to *t* = 25 min. Subsequently, initial conditions were recovered in the subsequent 5 min, which ran until the end of the assay (*t* = 35 min). The injection volume was 20 µL. UV detection of analytes was carried out at 254 nm. 

#### 2.2.5. Encapsulation Efficiency of Dexamethasone-Loaded Microspheres (Dx-MS)

Sterilized and non-sterilized microspheres (5 mg) were dissolved in 5 mL of dichloromethane. Then, 12 mL of ethanol was added to promote polymer precipitation. Samples were further centrifuged (5000 rpm, 10 min) and the supernatant was recovered and filtered (0.45 µm). Dexamethasone in the supernatant was quantified by HPLC as described in Section 2.2.4.

The encapsulation efficiency (EE%) was calculated as follows (Equation (2)):

(2) $$\mathrm{EE}\;\%=\frac{Actual\;drug\;content}{Theorical\;drug\;content}\;\times \;100.$$

#### 2.2.6. In Vitro Release Studies

Duplicates of 5 mg of microspheres of each batch (sterilized and non-sterilized) were suspended in 2 mL of phosphate-buffered saline (PBS, pH 7.4) isotonized with NaCl. Samples (protected from light) were placed in a shaker bath with a constant agitation speed of 100 rpm (Clifton Shaking Bath NE5, Nikel Electro Ltd., Weston-super-Mare, UK; Avon, London, UK) at 37 °C. At preset times (1 h, 24 h, 48 h, and once every 3 days until the end of the study), the microparticle suspensions were gently centrifuged (3000 rpm, 3 min), and 2 mL of the supernatant was recovered and replaced with the same volume of fresh PBS, to continue the release study and assure sink conditions. The remaining microparticles and the fresh release media were gently mixed in a vortex before being replaced again in the shaker bath. The concentration of dexamethasone in the supernatant samples was analyzed using the HPLC method described in Section 2.2.4. Both the cumulative release (% vs. t) plot and the release rate (%/day) plot, calculated as the amount of drug released at each time point divided by the number of days between sample point, are presented.

#### 2.2.7. Analysis of Drug Release Mechanism

Two model-dependent approaches were selected to characterize the dissolution profiles: the Korsmeyer–Peppas equation (Equation (3)) [31,32] and the Heller and Baker equation (Equation (4)) [33].

(3) $$\frac{Q_{t}}{Q_{\infty }}=K\cdot t^{n},$$

(4) $$\frac{dQ}{dt}=\frac{A}{2}\frac{\sqrt{2\cdot P_{0}\cdot e^{Kc\cdot t}\cdot C_{0}}}{t},$$

where, in Equation (3), *Q_t_*/*Q_∞_* represents the fraction of drug released at time *t*, *K*, is the kinetic constant of the release process, characteristic of the drug/polymer system, *t* is the release time, and *n* is the release exponent that describes the drug release mechanism. In Equation (4), *A* is the contact area, *P*_0_ is the initial drug permeability, *K_c_* is the first-order kinetic polymeric alteration rate constant, *C*_0_ is the initial drug concentration, and *t* is the time. In both cases, only the data points with less than 60% release were used.

Considering microspheres as a matrix, i.e., heterogeneous and porous systems that hydrate when in contact with the aqueous medium, the release profile of the drug will depend on both the diffusion rate of the active principle in the matrix and the rate of hydration of the polymer. If the erosion phenomena described within the mechanisms that affect this type of release are also considered, the kinetics that can be manifested in these studies can be complex. That is why, in this work, two models were selected that have proven to be useful to describe these complex systems. One of these treatments is that proposed by Korsmeyer et al. (Korsmeyer equation). According to this model, when polymeric materials in contact with a solvent are hardly modified or adapt quickly to the new situation, the predominant mechanism is Fick diffusion, with the transfer rate (dQ/dt) being inversely proportional to the square root of time, i.e., the value of *n* in the above equation is equal to 0.5. This situation is also known as case I transport. If the transfer rate is influenced both by the diffusion of the solute and by modifications in the reticular structure of the polymer (coupling of the solvent molecules or hydration, relaxation, or breakage of the polymer chains), then this value of *n* is greater than 0.5, and the below situations can be established.

Anomalous transport is where the release of the solute is controlled both by its diffusion and by the structural modification of the lattice. The value of *n* is then between 0.5 and 1. Transport case II and super-case II occur when the transfer rate is not controlled by diffusion mechanisms, but by the alterations that take place in the polymer structure (coupling, rupture, or adjustment), manifesting a kinetic that is good; it depends linearly on time (zero-order, *n* equal to 1), being called case II transport, and it can be of higher degree (*n* > 1), which in this case is called super-case II transport. Later, Ritger and Peppas described different values of *n* depending on the geometry of the devices. For spherical matrices, the corresponding extreme values of *n* are 0.43 and 0.85 [34,35].

Another of the equations proposed for those cases in which homogeneous polymer erosion processes are developed is that described by Heller and Baker, where the transfer rate corresponds to Higuchi’s basic approach, but introduces a variable permeability coefficient throughout time, which depends on the alteration in the polymer structure (internal erosion process), admitting that this last process responds to a kinetics of order one. This treatment can only be applied when the transfer rate increases with time (since an increase in the diffusion coefficient is admitted), and the results close to or after the time in which the maximum transfer rate is manifested should not be considered.

### 2.3. Sterilization of Microspheres

Dx-MS were subjected to ^60^Cobalt radiation at 25 kGy. Samples were placed into 2 mL glass vials before closing with rubber and an overseal closure. Vials were sterilized in dry ice to assure a low temperature (−78.5 °C) during the irradiation process [36].

After sterilization, microspheres were further characterized in terms of mean particle size and particle size distribution, morphological evaluation, polymer integrity, drug content, and in vitro release profiles using the protocols described above. The drug release kinetics was evaluated according to the model-dependent approaches already mentioned. Furthermore, the dexamethasone release profiles from sterilized and non-sterilized microspheres were further compared using a model-independent approach, by calculating the similarity factor (*f_2_*) (Equation (5)).

(5) $$f_{2}=50\times Log\left\{{\left[1+\left(1/n\right)\sum_{t=1}^{n}{\left|R_{t}-T_{t}\right|}^{2}\right]}^{-0.5}\times 100\right\},$$

where *Log* is the logarithm base 10, *n* is the number of observations, *R_t_* is the average percentage drug dissolved from the reference formulation, and *T_t_* is average percentage drug dissolved from the test formulation. 

### 2.4. In Vivo Studies

A total of 22 female pigmented rabbits weighing 2.5–3.5 kg were used. The procedure assured animal welfare, and it was approved by the University of Valladolid Ethics Committee on animal experimentation and welfare. Furthermore, the protocols herein comply with the ARVO Statement for the Use of Animals in Ophthalmology and Vision Research and are in accordance with the European Communities Council Directive (86/609/EEC). 

Before administration, animals were anesthetized by applying an intramuscular injection of 30 mg/kg of ketamine (Imalgene^®^ 1000, 100 mg, Merial Laboratorios S.A., Barcelona, Spain) and 6 mg/kg of xylazine (Rompun^®^ 2%, 2 g, Bayer AG, Barcelona, Spain). Pinna and pedal reflexes were used to monitor the level of anesthesia. Topical anesthesia was applied on the eye prior to the procedure via topical instillation of tetracaine chlorhydrate (Colircusí Anestésico Doble^®^ 0.05%, Alcon Cusí, Barcelona, Spain).

Microspheres were injected at the posterior superotemporal juxtascleral area (sub-Tenon). Briefly, the upper eyelid was retracted, and the superior rectus muscle was rotated nasally to expose the superior temporal quadrant of the eye. The conjunctiva was held with toothed forceps, and a buttonhole was cut with Westcott scissors. Afterward, a blunt dissection was conducted in order to introduce a metal cannula, 19G (model Stevens, Magnolia, NJ, USA), under the Tenon’s capsule. The cannula was fixed to a syringe preloaded with the microparticle suspension, and the formulation was slowly delivered into the sub-Tenon’s space. Finally, the cannula was carefully removed. In order to avoid any post-injection leakage, a sterile cotton-tipped applicator was used to gently press over the insertion area. Tobramycin ointment (Tobrex^®^ 3 mg/g, Alcon Cusí, Barcelona, Spain) was applied at the site of injection. On day 0, a sub-Tenon injection of 5 mg of Dx-PLGA microspheres (dose: 828 µg of dexamethasone) in PBS (100 µL) was applied to the left eye of each animal. The right eye was used as the control and received 100 µL of PBS. A clinical evaluation was performed in all eyes until the end of the study.

A physical examination and scored ophthalmic evaluation of the animals were performed at the time of injection and 1, 7, 14, 28, and 42 days later. Anterior pole evaluation was performed by slit-lamp biomicroscopy (Kowa SL-15; Kowa Optimed Inc., Torrance, CA, USA) following the scored method described by Hackett and McDonald [23]. Posterior pole evaluation was performed by inverted image ophthalmoscopy (Keeler Ltd., Windsor, UK) with non-contact lenses and pharmacological midriasis with Tropicamide^®^ (Colircusí Tropicamida^®^ colirio; Alcon Cusí S.A.). Intraocular pressure (IOP) was scored by contact tonometry (Tono-Pen VetTM Tonometer; Reichert Inc., Depew, NY, USA). The following clinical signs were evaluated: conjunctival congestion, swelling and discharge, aqueous flare, light reflex, iris involvement, pannus, vitreous opacity, vascular congestion, vitreous and retinal hemorrhage, retinal detachment, and intraocular pressure (Table 1).

Two animals were euthanized at day 42 post injection, and the eyes were submitted for histological processing and microscopic evaluation by hematoxylin and eosin staining. The tissue response in the areas of the injection site was evaluated for quantity of inflammation (− absence; + mild; ++ moderated; +++ severe; +/− does not fall into one of the defined categories), predominant cell type of the inflammatory infiltrate, presence of foreign body giant cell reaction (+ present; − absent), and presence of residual microspheres (+ present; +/− focal, occasional; − absent). 

### 2.5. Statistical Analysis

The data were represented as means ± SD for *n* values. Statistically significant differences were calculated by one-way analysis of variance (ANOVA). The differences were considered significant at *p* < 0.05.

## 3. Results

### 3.1. Microsphere Preparation and Characterization

Data concerning the preparation parameters and the characteristics of the formulations are compiled in Table 2. As can be observed, the production yield was independent of the emulsification speed (*p* = 0.9905) with values around 60% in all cases. 

**Figure 1:**
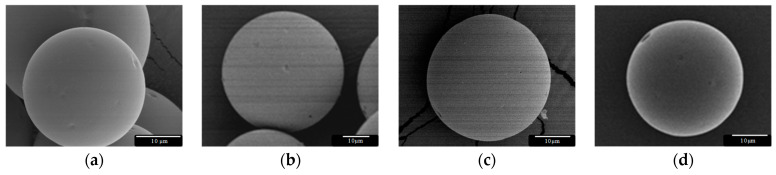
Dexamethasone (Dx)-loaded poly(lactic-*co*-glycolic) acid (PLGA) microspheres (MS, 20–53 µm) prepared using the oil-in-water (O/W) emulsion solvent evaporation technique at (**a**) 5500 rpm, (**b**) 8500 rpm, (**c**) 9500 rpm, and (**d**) 10,500 rpm.

As expected, this parameter did clearly influence the particle size distribution, showing a shift to smaller particle sizes, especially when using emulsification speeds higher or equal to 9500 rpm. In all cases, particles exhibited a spherical shape with only some small pores and no crystals on the surface, as shown by SEM (Figure 1).

In Table 3, Dx loading of the two fractions of interest (53–38 µm and 38–20 µm) is presented, as well as the percentage encapsulation efficiency (EE%). Considering the fraction with the biggest particle size (53–38 µm), the highest encapsulation efficiency was achieved by formulation 4 with values of 103.2% ± 0.4%. The values of the two granulometric fractions in the case of formulation 3 resulted in nonsignificant differences (*p* = 0.896), and the complete 53–20 µm fraction was selected for the in vivo studies. 

### 3.2. Sterilization of Dx PLGA Microspheres

**Figure 2:**
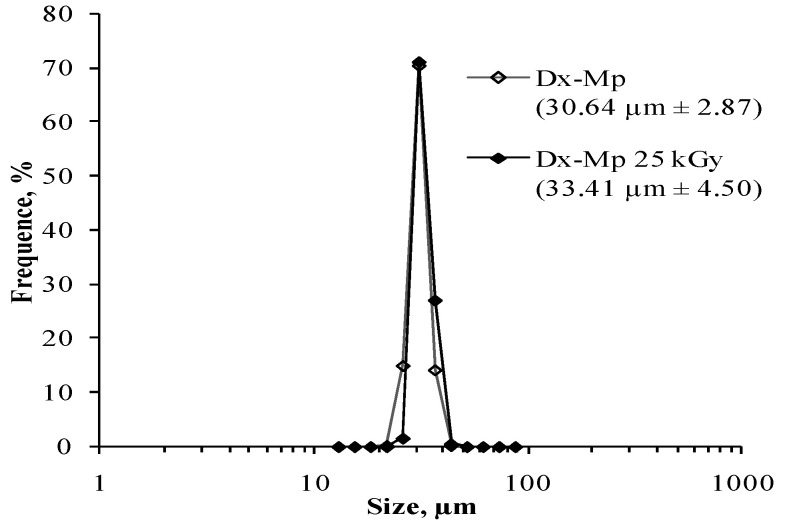
Particle size distribution of dexamethasone-loaded microspheres (53–20 µm): ◊ before and ♦ after sterilization.

**Figure 3:**
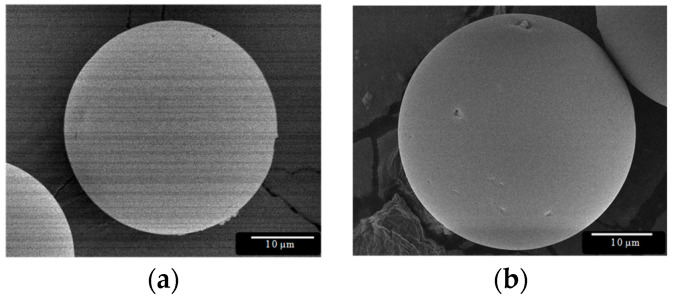
SEM pictures of dexamethasone-loaded PLGA microspheres: (**a**) before and (**b**) after γ-irradiation (applied dose: 25 kGy).

The size and morphology of the Dx-MS was not influenced by the sterilization procedure (*p* = 0.540), as shown in Figure 2 and Figure 3. The product retained its physical characteristics, and particles were free-flowing without showing clumping and/or aggregation behavior. 

**Figure 4:**
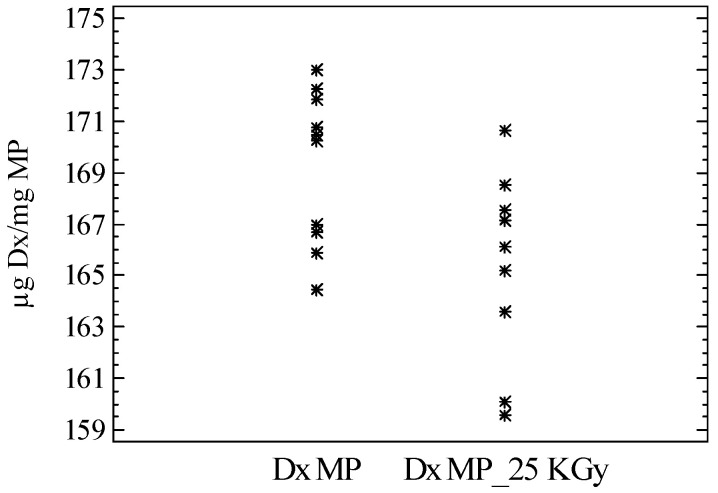
Dexamethasone loading in the microparticles (µg Dx/mg MS) before (Dx MS) and after sterilization (Dx-MS 25 kGy) (*n* = 10).

**Figure 5:**
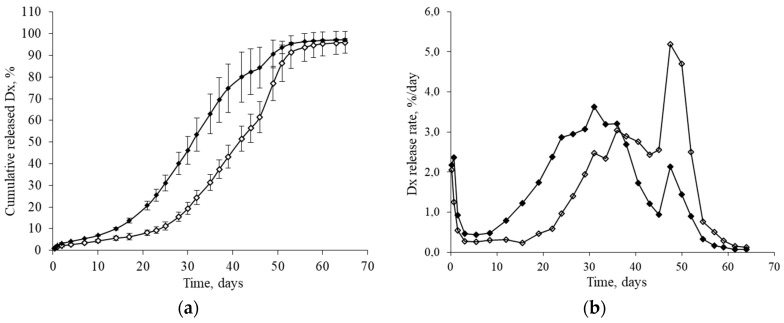
Release profile of dexamethasone from non-sterilized (◊) and sterilized (♦) PLGA microspheres (53–20 µm, prepared at 9500 rpm). (**a**) Cumulative release (%) and (**b**) release rate (%/day). Data points (± standard deviations) (*n* = 10).

The drug content and release characteristics of 10 independent batches of sterilized microspheres were also analyzed. According to the results, dexamethasone content remained unaltered with a mean value of 165.6 ± 3.6 µg Dx/mg MS (Figure 4). These systems also provided a controlled release of the drug for 9 weeks (Figure 5a,b). 

The release profile of Dx from the PLGA MS exhibited sensible changes after sterilization (Figure 5a,b). For example, the time necessary to release 50% of loaded dexamethasone was reduced to 12 days for gamma-irradiated samples as compared with non-sterilized particles. The similarity factor f_2_ was selected as a model-independent method to compare the two release profiles, obtaining an f_2_ value of 35.5, lower than the similarity limit (*f_2_* = 50), confirming that the changes in the release profile were due to the sterilization process. These changes in the Dx release profile after gamma-irradiation were attributed to the induction of ester bond cleavage and the resulting decrease in the polymer molecular weight [26,37,38].

**Figure 6:**
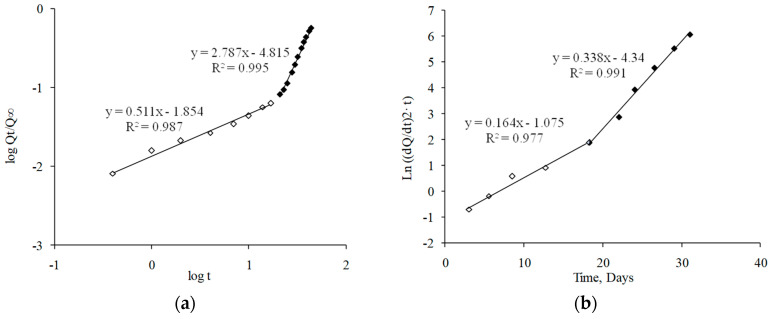

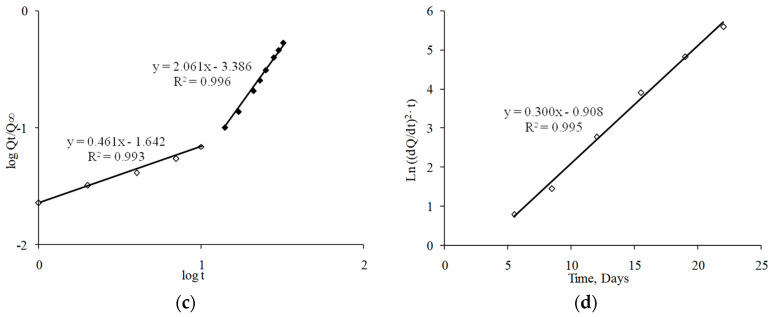
Kinetic fitting of dexamethasone release data: (**a**) Korsmeyer−Peppas model before sterilization; (**b**) Heller and Baker model before sterilization; (**c**) Korsmeyer−Peppas model after sterilization; (**d**) Heller and Baker model after sterilization.

As can be observed in Figure 6a,c, experimental data fit the Korsmeyer–Peppas equation showing a biphasic behavior. Determination coefficient and “*n*” values are compiled in Table 4. In accordance with this model, the first phase of the release (0–17 days) was characterized by an anomalous transport (*n* = 0.51), indicating that not only drug diffusion, but also polymeric matrix changes controlled the release of dexamethasone [39]. In the second phase (17 days until the end), the strong alterations produced on the polymeric matrix structure might control the drug release (*n* > 0.85), according to a Super Case II transport. 

Figure 6b,d show the fitting of experimental data for the first 32 days of the release assay to the Heller and Baker model, considering that a homogeneous polymer erosion occurs during the drug release. According to this model, two polymeric change rates occurred (data shown in Table 4). The first phase (approximately the first 17 days of the study) was characterized by a slow polymer alteration (K_c_ = 0.16 day^−1^). In the second phase, an increase in the polymeric alteration rate was achieved (K_c_ = 0.34 day^−1^). 

**Figure 7:**
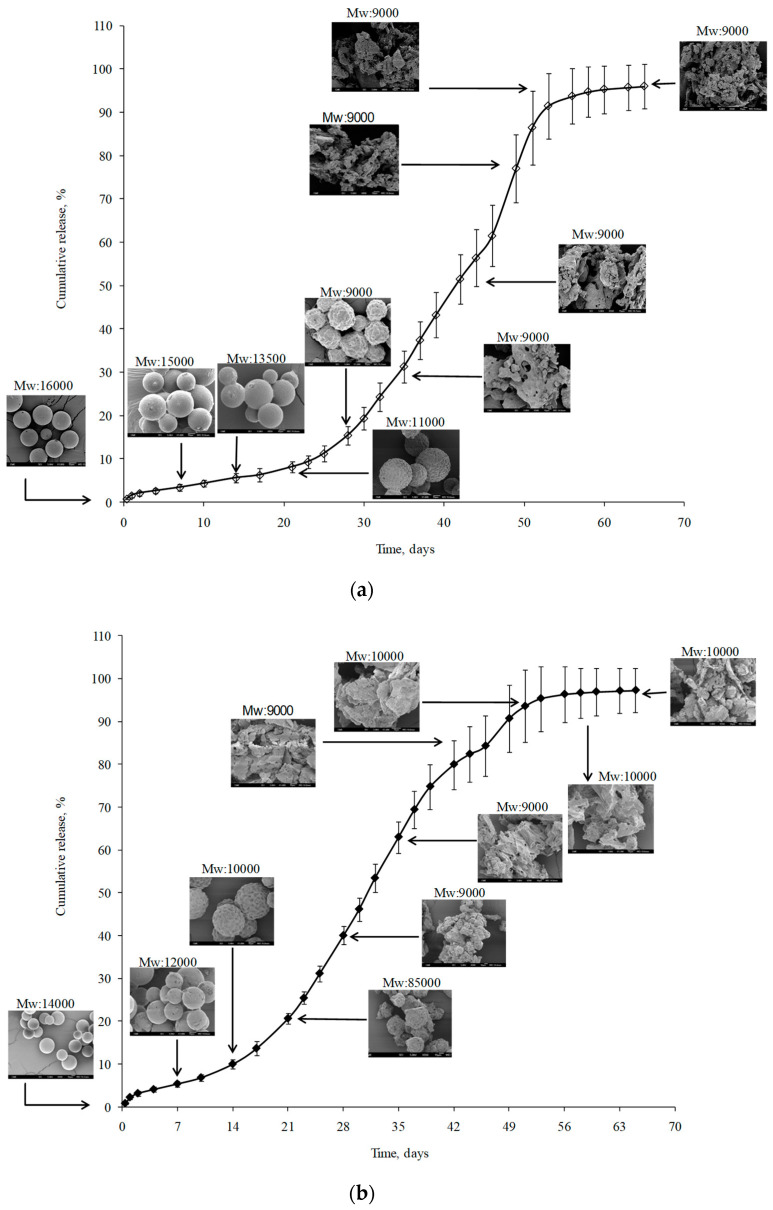
PLGA molecular weight (gel permeation chromatography (GPC) data) and microsphere morphology (SEM pictures) during the in vitro release assay of non-sterilized (**a**) and sterilized (**b**) microspheres.

In order to complete and integrate the information obtained from the kinetic evaluation of the drug release profiles, both the polymer molecular weight (M_W_) and the morphological aspects of microspheres were monitored during the release study (Figure 7). Effectively, during the first 15–17 days, microspheres did not appreciably change their external morphology and, therefore, exhibited a spherical shape over this time period. At the same time, only a moderate reduction in PLGA M_W_ (15%) was observed. This behavior should be in concordance with the anomalous transport proposed after Korsmeyer–Peppas fitting and with the low polymer erosion rate observed after Heller and Baker treatment. During weeks 3 and 4, microspheres maintained their spherical structure; however, their surfaces started to deteriorate more. This may be explained by the higher polymer erosion rate observed (Table 4), which was confirmed by the reduction in the polymer M_W_ (45% of the initial value). No further M_W_ reduction was observed until the end of the study. However, according to the SEM pictures, in the fifth week of the release, the inner microsphere structure appeared to be so weak that particles disintegrated, which may be the origin of the Super Case II transport observed according to Korsmeyer–Peppas fitting in the second phase of the study.

The kinetic analysis of drug release data following both Korsmeyer–Peppas and Heller and Baker models showed changes related to the polymer matrix modifications due to sterilization. On one hand, the Korsmeyer·Peppas studies (Table 4, Figure 6c) showed a more extended Super Case II transport kinetic starting 1 week before (at day 10 instead of day 17) for sterilized MS. Furthermore, the Heller and Baker fitting (Table 4, Figure 6d) behaved in a monophasic manner, with a high polymer alteration rate constant (0.30 day^−1^). Additionally, both the PLGA molecular weight measurement and the SEM pictures of sterilized microspheres during the release process confirmed the kinetic results, showing a higher erosion of the polymer both on the surface (surfaces began to deteriorate after 2 weeks of study) and in the inner structure; therefore, particles disintegrated after 3 weeks (Figure 7b).

### 3.3. In Vivo Evaluation

Table 5 shows the results of the ophthalmic clinical signs evaluated after sub-Tenon injection. Among the clinical signs evaluated, only conjunctival congestion was observed.

Experimental eyes presented different degrees (minimal or mild) of conjunctival congestion at 1 day (19/20) and at 7 days (7/15); however, in all cases, conjunctival congestion disappeared at 14 days post injection. In addition, conjunctival congestion was present in the control eye of 25% of animals at 1 day (5/20). As hypothesized by other authors, the conjunctival congestion observed in both eyes (treated and untreated) could be due to the sub-Tenon injection itself [40]. 

No other clinical changes were observed in terms of conjunctival discharge, conjunctival swelling, aqueous flare, light reflex modification, iris involvement, surface of cornea cloudiness, pannus, fluorescein staining, loss of lens transparency, vitreous opacity, vascular congestion, vitreous and/or retinal hemorrhage, and/or retinal detachment. According to these results, microspheres were well tolerated in the short term after periocular injection.

**Figure 8:**
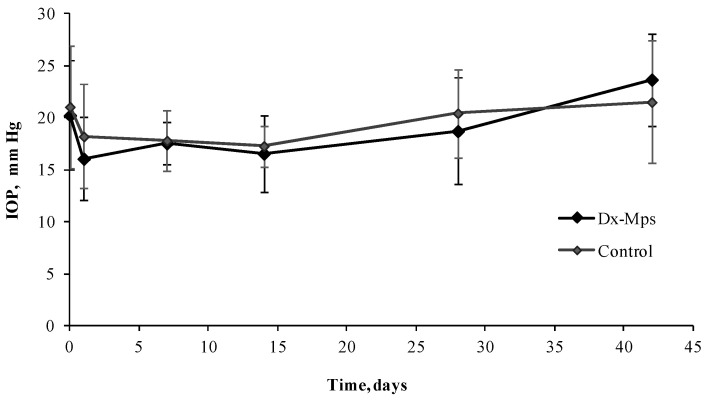
Intraocular pressure (IOP) mean values (± standard error of the mean (SEM)) obtained at different times of the study: before injection, and 1, 7, 14, 28, and 42 days after injection, for control and eyes that received a sub-Tenon injection of Dx-MS. No significant differences were found between groups (*p* > 0.05).

In the present study, intraocular pressure (IOP) remained unchanged with mean values of 19.4 ± 1.8 mmHg (control eyes) and 18.8 ± 2.8 mmHg (experimental eyes) during the 45 days of follow-up. The statistical analysis of IOP values showed no significant differences (*p* > 0.05) between groups (Figure 8).

### 3.4. Histological Analysis

**Figure 9:**
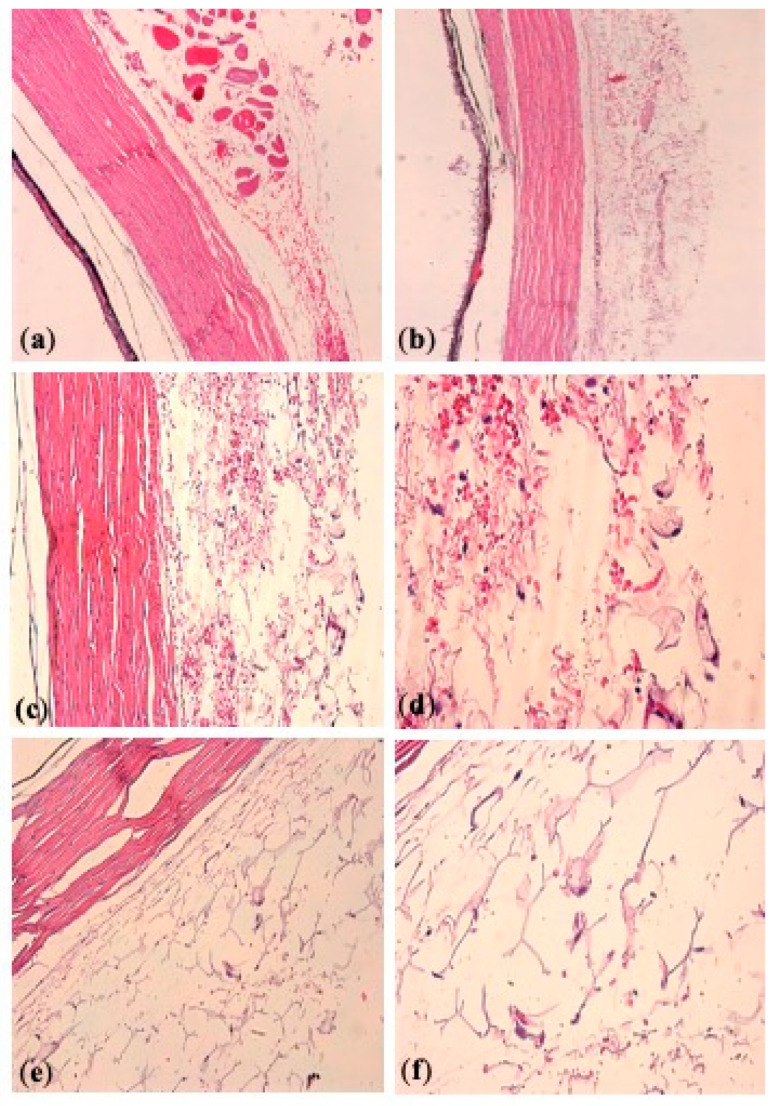
Histopathologic analysis of episcleral tissue injection site, after sub-Tenon injection of Dx-microspheres in phosphate-buffered saline (PBS) (left eye, **a**,**b**) and control eye (right eye, **c**–**f**). (**a**,**b**) Untreated eyes of two different rabbits, showing absence of tissue reaction; (**c**,**d**) Dx-microparticle-treated eye (rabbit 1), showing presence of occasional foamy macrophages and no microsphere residues; (**e**,**f**) Dx-microsphere-treated eye (rabbit 2), showing presence of single foamy macrophages and scattered occasional microspheres residues. Magnification: 10× (**a**,**b**); 20× (**c**,**e**); 40× (**d**,**f**).

Four eyes (two experimental and two control eyes) were subjected to histopathology studies at 42 days after injection (Figure 9, Table 6). After dosing with Dx-MS, ocular tissues close to the injection site showed occasional macrophages. As no inflammatory signs were noted in the eyes belonging to the control group, the immunological reaction observed in experimental eyes might be related to the presence of microspheres at the administration site. Additionally, scattered occasional residues of microspheres were observed in one experimental eye. 

## 4. Discussion

Current developments in the therapy of ophthalmic chronic diseases are directed toward the use of new drug delivery systems, with the objective to allow sustained therapeutic levels of drugs at ocular target sites. In this sense, biodegradable microspheres have been demonstrated to provide long-term delivery of drugs, and their utility in ophthalmology has been previously reported [3,15,41,42,43,44]. One of the main advantages of these systems is that they can be administered as a conventional injection using a small=gauge needle. Furthermore, depending on the patients’ needs, a proper dose of the active substance can be adjusted by administering a specific amount of microspheres, allowing a personalized therapy. The most effective route of administration to achieve appropriate drug levels in the posterior segment of the eye is intravitreal injection. However, the use of this route in chronic therapies has potential side-effects, such as retinal detachment or endophthalmitis, and other alternatives are under study. In this experimental work, biodegradable PLGA microspheres loaded with the anti-inflammatory dexamethasone were designed to allow a long-term release of the drug by periocular administration, in line with previous studies in which authors demonstrated that this drug can diffuse through the sclera and can be detected in the retina and vitreous [21,22,23].

Even though PLGA is a Food and Drug Administration (FDA)-approved polymer to be used in devices, it is desirable to minimize the amount of PLGA administered to the eye. This can be achieved by fabricating PLGA microparticles that exhibit a high drug-loading capacity. In this sense, all formulations and size fractions presented in this work showed excellent encapsulation characteristics, with high drug loadings in all the assayed conditions). These results are in accordance with those reported by other authors [45]. Another desirable attribute of microparticles is that they should exhibit a particle size that is small enough to pass through a cannula, needed to inject these formulations in the form of aqueous suspensions. As formulation 3 did not show significant differences between the two granulometric fractions assayed (53–38 µm and 38–20 µm), the complete 20–53 µm fraction, with a mean particle size of 31 ± 3 µm, was chosen as the most suitable for injection.

Many authors have proposed a gamma-irradiation sterilization process as one of the least aggressive and more suitable sterilization approaches for PLGA microspheres with no significant changes in MS properties [15,26,28,29]. In the present work, the surface morphology and particle size distribution of microspheres remained unaltered after the sterilization process under the described conditions. However, changes in the release profile of dexamethasone were observed in sterilized batches, which might be attributed to the induction of ester-bond cleavage and the resulting decrease in polymer molecular weight [26,37,38]. It is well known that a decrease in M_W_ may have an important effect on the mobility of the polymer chain and, hence, on the free volume available for water and drug diffusion [28]. Effectively, in this study, a reduction in the PLGA molecular weight by 11% was quantified after irradiation. These results are in agreement with other works, wherein a loss in M_W_ was documented for PLGA MS after gamma sterilization [30,37,46].

Ideally, drug delivery devices will maintain therapeutic concentrations locally for months. Poly(lactic acid), poly(glycolic acid), and their copolymers degrade safely via hydrolysis into natural products of lactic and glycolic acids, which are metabolized into CO_2_ and water [47]. PLGA is preferred for microparticle preparation in part because of its slow and reproducible degradation rate. PLGA has been used in several FDA-approved parenteral drug applications that are currently on the market, with one of them being used in ophthalmology, for intravitreal administration [7]. Although several authors have demonstrated that the in vitro/in vivo correlation is not optimal for ocular tissues [47], in vitro release studies remain a preliminary step necessary to determine the potential suitability of developed formulations. In the present work, changes in the release profile were detected and attributed to the induction of ester-bond cleavage and the resulting decrease in polymer molecular weight after the gamma irradiation [26,37,38]. However, the results shown in this work demonstrate that the developed microspheres were able to control the release of dexamethasone until even 9 weeks after their sterilization, a requirement needed for the use of these systems in vivo.

Microspheres were well tolerated after periocular injection. Among the clinical signs evaluated, only conjunctival congestion was observed. As hypothesized by other authors, the conjunctival congestion observed in both eyes (treated and untreated) could be due to the sub-Tenon injection itself [40]. In fact, Saishin et al. (2003) observed that, 20 days after periocular administration of unloaded PLGA–glucose microspheres and PKC412 microspheres (25% or 50%) in pigs, mild conjunctival congestion was similarly observed among the three groups, and there were no discernible signs of inflammation or irritation. In addition, Amrite et al. [48] did not find any signs of inflammation, including redness or edema, after a single subconjunctival injection of PLGA microspheres loaded with celecoxib in rats.

Concerning the nature of the administered drug, different adverse events can occur after corticosteroid therapy. The most common adverse reactions are cataract formation and elevated intraocular pressure [10,49], as well as the promotion of ocular herpes simplex virus reactivation [50]. In the present work, no clinical signs of cataract formation were denoted during the 6 weeks of observation. However, it is important to remark that the treatment-related cataract formation may take longer periods to become apparent [10]. The increase in IOP after administration of corticosteroids is of particular concern [10,51]. Whereas this increment reverts to normal values when the treatment is finished, the glaucomatous damage of the optic nerve head is irreversible, and patients need an ocular hypotensive treatment for the duration of the corticosteroid treatment in order to avoid permanent damage. This fact is of special importance in chronic treatment with steroids. Effectively, some authors showed glaucomatous symptoms associated with the ocular administration of corticosteroids due to the increment of IOP, typically after systemic and topical administration [52]. In the present study, IOP remained unchanged during the assay (42 days) with a mean after injection of 19.4 ± 1.8 mmHg (control eye) and 18.8 ± 2.8 mmHg (experimental eye). The statistical analysis of IOP values showed no significant differences (*p* > 0.05) between groups. 

Scattered occasional residues of microspheres were observed in one experimental eye. According to the previous extensive in vitro evaluation of microspheres presented in this work, once administered, PLGA microspheres might undergo progressive polymer hydrolysis while remaining visible for at least 6 weeks. It is, thus, not uncommon to find small portions of polymeric material in histological samples. These small portions might suffer progressive degradation until disappearance, with no observable tissue damage, according to previous tolerance studies where PLGA microspheres were intravitreally [15] or periocularly [24] administered, and this was even noted with the use of the more hydrophobic PLGA (85:15), which presents a lower degradation rate [53].

A total of 42 days after periocular injection, Dx-MS were still detected in the sub-Tenon space by histopathology studies, indicating that they were able to properly reach the injection site. No clinical signs of inflammation were observed during the study, although it is true that particles were loaded with an anti-inflammatory drug that could mask this symptom. On the contrary, an immunological reaction was observed at the site of administration. This “foreign body reaction” resulted analogous to that observed after the previously described intravitreal injection of 10 mg of PLGA microspheres into rabbits [54], and similar to that described for microspheres injected intramuscularly in rabbits, which gradually decreased with time [55]. 

The findings presented in this work are promising since, to date, research has shown limited data on creating a proficient system for delivering small-particle-size drugs (such as dexamethasone) periocularly over a prolonged period of time. By encapsulating dexamethasone in PLGA microspheres with the method optimized here, it has been demonstrated that a slow and sustained drug delivery can be achieved in a local environment, resulting in long-term delivery of the drug for over 9 weeks. 

## 5. Conclusions

The periocular administration of microparticulate systems can be considered as a good alternative to overcome the limitation of the frequent intravitreal administration of drugs. Dx-MS developed in this work represent a suitable formulation for sub-Tenon administration. Particles exhibited a high encapsulation efficiency, as well as prolonged and sustained release rates, and they were resistant to gamma sterilization. Microspheres were well tolerated after in vivo administration with no increase in IOP for at least 42 days. Although prolonged efficacy and tolerance studies are needed to study the effect of these systems in more depth, this formulation is presented as a potential alternative for the long-term delivery of ophthalmic drugs to the back of the eye.

## Figures and Tables

**Table 1:** Ophthalmic evaluation using scored method.

Anterior Pole Evaluation [23]		
Conjunctival congestion	0	Normal, blanched to reddish pink conjunctiva
	1 to 3	From reddish color confined to the palpebral conjunctiva to dark red color of both palpebral and bulbar conjunctiva
Conjunctival swelling	0	Normal, no swelling
	1 to 4	From swelling above normal without eversion of the lids to swelling with pronounced eversion of the lids
Conjunctival discharge	0	Normal, no discharge
	1 to 3	From discharge above normal and restricted to the inner portion of the eye to discharge flowing over the eyelids
Aqueous flare	0	Absence of visible light beam in the anterior chamber
	1 to 3	From Tyndall effect barely discernible to Tyndall beam easily discernible
Light reflex	0	Normal pupillary response
	1 or 2	From sluggish to fixed pupillary response
Iris involvement	0	Normal iris without hyperemia
	1 to 4	From minimal injection of secondary vessels to marked injection of secondary and tertiary vessels with marked swelling of the iris stroma
Cornea	0	Normal
	1 to 4	From some loss of transparency to marked loss of transparency of the entire thickness of the corneal stroma
Surface of cornea cloudiness	0	Normal
	1 to 4	From 1–25% to 76–100% area of stromal cloudiness
Pannus	0	Absence
	1 or 2	From vascularization without vessels invading the cornea to vessels invading 2 mm or more of the cornea
Fluorescein staining	0	Absence
	1 to 4	From slight and confined to a small focus to extreme fluorescein staining
Lens	Normal/abnormal	
**Posterior Pole Evaluation**		
Vitreous opacity	Yes/No	
Vascular congestion	Yes/No	
Vitreous and/or retinal hemorrhage	Yes/No	
Retinal detachment	Yes/No	

**Table 2:** Production yield (Y) and particle size distribution of formulations elaborated at different homogenization speeds (HS) (*n* = 3).

Formulation	HS (rpm)	Y (%)	Size Distribution (%)				
			>106 µm	106–53 µm	53–38 µm	38–20 µm	20–2 µm
1	5500	59.4 ± 1.4	9.8 ± 2.8	48.1 ± 2.2	16.9 ± 0.8	16.8 ± 0.2	8.4 ± 0.6
2	8500	60.4 ± 3.0	-	19.5 ± 5.2	32.0 ± 1.3	32.7 ± 2.9	15.8 ± 1.1
3	9500	60.2 ± 2.8	-	18.1 ± 1.7	28.8 ± 2.6	33.3 ± 1.2	19.8 ± 3.7
4	10,500	59.9 ± 1.2	-	16.0 ± 3.7	26.7 ± 0.6	34.1 ± 2.3	23.3 ± 1.1

**Table 3:** Drug loading (µg Dx/mg MS) and percentage encapsulation efficiency (EE%) for the fractions of interest of each formulation (*n* = 3).

Formulation	µg Dx/mg MS		EE (%)	
	53–38 µm	38–20 µm	53–38 µm	38–20 µm
1	154.3 ± 4.7	131.1 ± 4.7	92.5 ± 3.0	78.6 ± 2.7
2	161.4 ± 2.5	150.1 ± 4.9	86.7 ± 1.4	89.9 ± 2.9
3	164.0 ± 6.0	162.7 ± 7.2	98.1 ± 3.6	97.3 ± 4.3
4	172.0 ± 0.9	146.7 ± 1.8	103.2 ± 0.4	94.0 ± 1.1

**Table 4:** Values of kinetic parameters of drug release profiles before and after sterilization. The value of “*n*” of Korsmeyer–Peppas model (95% confidence level, CL), “K_c_” of Heller and Baker model, and coefficients of determination (*r^2^*).

Drug Release	Korsmeyer-Peppas Model			Heller and Baker Model		
	Phase	*r^2^*	*n* (CL)	Phase	*r^2^*	K_c_ (days^−1^)
Dx-MS	1	0.987	0.51 (0.46–0.56)	1	0.977	0.16
	2	0.995	2.79 (2.66–2.91)	2	0.991	0.34
Dx-MS, 25 kGy	1	0.993	0.46 (0.41–0.51)	1	0.995	0.30
	2	0.996	2.06 (1.96–2.16)			

**Table 5:** Summary of ophthalmic clinical observations (anterior and posterior segment). Rabbits received a single sub-Tenon injection of Dx-loaded microspheres in the left eye (LE). The right eye (RE) was used as the control.

Sign	Prescreen		1 day		7 days		14 days		28 days		42 days	
	RE	LE	RE	LE	RE	LE	RE	LE	RE	LE	RE	LE
Conjunctival congestion	1/20	2/20	5/20	19/20 *	0/15	7/15 **	0/11	0/11	0/7	0/7	0/6	0/6
Conjunctival discharge	0/20	0/20	0/20	0/20	0/15	0/15	0/11	0/11	0/7	0/7	0/6	0/6
Conjunctival swelling	0/20	0/20	0/20	0/20	0/15	0/15	0/11	0/11	0/7	0/7	0/6	0/6
Aqueous flare	0/20	0/20	0/20	0/20	0/15	0/15	0/11	0/11	0/7	0/7	0/6	0/6
Light reflex	0/20	0/20	0/20	0/20	0/15	0/15	0/11	0/11	0/7	0/7	0/6	0/6
Iris involvement	0/20	0/20	0/20	0/20	0/15	0/15	0/11	0/11	0/7	0/7	0/6	0/6
Surface of cornea cloudiness	0/20	0/20	0/20	0/20	0/15	0/15	0/11	0/11	0/7	0/7	0/6	0/6
Pannus	0/20	0/20	0/20	0/20	0/15	0/15	0/11	0/11	0/7	0/7	0/6	0/6
Fluorescein staining	0/20	0/20	0/20	0/20	0/15	0/15	0/11	0/11	0/7	0/7	0/6	0/6
Lens	0/20	0/20	0/20	0/20	0/15	0/15	0/11	0/11	0/7	0/7	0/6	0/6
Vitreous opacity	0/20	0/20	0/20	0/20	0/15	0/15	0/11	0/11	0/7	0/7	0/6	0/6
Vascular congestion	0/20	0/20	0/20	0/20	0/15	0/15	0/11	0/11	0/7	0/7	0/6	0/6
Vitreous and retinal hemorrhage	0/20	0/20	0/20	0/20	0/15	0/15	0/11	0/11	0/7	0/7	0/6	0/6
Retinal detachment	0/20	0/20	0/20	0/20	0/15	0/15	0/11	0/11	0/7	0/7	0/6	0/6

* Animals showed minimal conjunctival congestion, except two animals that presented mild congestion. ** Animals showed minimal conjunctival congestion, except one animal that presented mild congestion.

**Table 6:** Scored histopathological evaluation at 42 days after sub-Tenon injection of Dx-microspheres in PBS (left eye, LE) and control eye (right eye, RE).

Eyes Evaluated	Inflammation at the Injection Site	Presence of Foreign Body Giant Cell Reaction	Presence of Microparticles Residues
Rabbit 1 and 2 RE (control)	− (Absent)	− (Absent)	− (Absent)
Rabbit 1 LE (Dx-MS)	− (Absent)	− (Absent)	− (Absent) No significant residues; occasional foamy macrophages
Rabbit 2 LE (Dx-MS)	− (Absent)	− (Absent)	+/− (focal) Scattered occasional residues; single foamy macrophage
